# The geographical distribution of scorpions, implication of venom toxins, envenomation, and potential therapeutics in Southern and Northern Africa

**DOI:** 10.1093/toxres/tfae118

**Published:** 2024-08-04

**Authors:** Isac G Mabunda, Nodji K Zinyemba, Shanelle Pillay, Benedict C Offor, Beric Muller, Lizelle A Piater

**Affiliations:** Department of Biochemistry, Corner of Kingsway and University Road, Auckland Park Campus, University of Johannesburg, Auckland Park, 2006, Gauteng, South Africa; Department of Biochemistry, Corner of Kingsway and University Road, Auckland Park Campus, University of Johannesburg, Auckland Park, 2006, Gauteng, South Africa; Department of Biochemistry, Corner of Kingsway and University Road, Auckland Park Campus, University of Johannesburg, Auckland Park, 2006, Gauteng, South Africa; Department of Biochemistry, Corner of Kingsway and University Road, Auckland Park Campus, University of Johannesburg, Auckland Park, 2006, Gauteng, South Africa; South Africa Venom Suppliers cc, 41 Louis, Trichardt 0920, South Africa; Department of Biochemistry, Corner of Kingsway and University Road, Auckland Park Campus, University of Johannesburg, Auckland Park, 2006, Gauteng, South Africa

**Keywords:** venom, antivenom, toxins, scorpion

## Abstract

Scorpions are predatory arachnids whose venomous sting primarily affects people in tropical and subtropical regions. Most scorpion stings can only cause localized pain without severe envenomation. Less than one-third of the stings cause systemic envenoming and possibly lead to death. About 350,000 scorpion stings in Northern Africa are recorded yearly, resulting in about 810 deaths. In Eastern/Southern Africa, there are about 79,000 stings recorded yearly, resulting in 245 deaths. Farmers and those living in poverty-stricken areas are among the most vulnerable to getting stung by scorpions. However, compared to adults, children are at greater risk of severe envenomation. Scorpion venom is made up of complex mixtures dominated by peptides and proteins that confer its potency and toxicity. These venom toxins have intra- and interspecies variations associated with the scorpion’s habitat, sex, diet, and age. These variations alter the activity of antivenoms used to treat scorpion sting envenomation. Thus, the study of the proteome composition of medically important scorpion venoms needs to be scaled up along their geographical distribution and contributions to envenomation in Southern and Northern Africa. This will help the production of safer, more effective, and broad-spectrum antivenoms within these regions. Here, we review the clinical implications of scorpion sting envenomation in Southern and Northern Africa. We further highlight the compositions of scorpion venoms and tools used in scorpion venomics. We discuss current antivenoms used against scorpion sting envenomation and suggestions for future production of better antivenoms or alternatives. Finally, we discuss the therapeutic properties of scorpion venom.

## Graphical Abstract

**Graphical Abstract:**
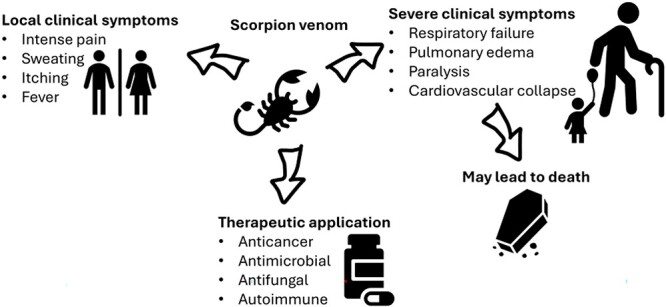

## Introduction

Scorpions belong to the predatory arachnid class of arthropods whose sting causes morbidity and mortality in many parts of the globe. Nearly 2.5 billion people are at risk of being stung by scorpions in several areas such as South Africa, northern-Saharan Africa, Near and Middle East, Sahelian Africa, Mexico and South Latin America, South India, and eastern Andes.^1^ The medically important scorpions are mainly those from the Buthidae family, which comprises *Androctonus, Buthus, Buthotus, Leiurus, Mesobuthus*, and *Parabuthus*, endemic to Asia, India, the Middle East and North Africa.^2^^,^^3^ In comparison, *Tityus* species are found mainly in South America and the Caribbean, while *Centruroides* species are found in Mexico, Central America and Southwest of the United States.^3–5^ Even though many of these species belong to the family Buthidae*,* other families, including Scorpionidae and Hemiscorpiidae, also have medically important species.^5^

Scorpion envenomation or scorpionism is a public health risk in tropical and subtropical regions worldwide. About less than one-third of scorpion stings cause systemic envenomation, eventually leading to death if not adequately treated.^6^ Scorpion stings and death can be prevented if robust safety awareness, precautions, and swift treatment processes are applied. Unfortunately, little attention is given to the morbidity and mortality rates caused by scorpion envenomation, especially in underdeveloped countries where venomous stings are under-reported.^1^ The estimated annual global scorpion envenomation incidence and mortality reported up to 1.5 million envenomings, leading to about 2,600 deaths,^6^ although these numbers are outdated. Equally, there are about 350,000 scorpion stings and 810 deaths in Northern Africa, while Eastern/Southern Africa has about 79,000 scorpion stings and 245 deaths.^7^ After Northern Africa, higher incidences were observed in Asia (250,000 stings and 645 deaths), Mexico (250,000 stings and 75 deaths), and the Near/Middle East (146,500 stings and 796 deaths). These numbers might have significantly increased since this available data was in 2008. Data on scorpion stings and envenomation collected by Tygerberg Poison Information Center (TPIC) South Africa between 2005 and 2014 showed that the Western Cape had the highest incidence.^8^ The incidence of scorpion stings is higher in adults but the severity of the envenomation is mostly experienced by children.^7^ It is believed that as time goes on, the number/cases of scorpion envenomation will increase due to climate change, population growth and also the expansion of urban which will increase the human interaction with these scorpions.^7^^,^^9^

Scorpion stings and envenoming, unlike snakebite envenoming is yet to be included as a neglected tropical disease (NTD) due to its current low mortality reports.^10^ The World Health Organization (WHO) Strategic and Technical Advisory Group for Neglected Tropical Diseases (STAG-NTD) argues and resolves it is yet to meet the requirement for NTD.^10^ Even though the number of scorpion envenomation-related deaths is significantly less compared to those caused by snakebite envenomation, it still imposes life-threatening health challenges to people in tropical and subtropical regions. Understanding the geographical localization of medically important scorpions in Southern and Northern Africa and their toxin profile will help in the development of safer, more effective, and broad-spectrum antivenom useful in the treatment of scorpion envenomation in these regions.

**Fig. 1:**
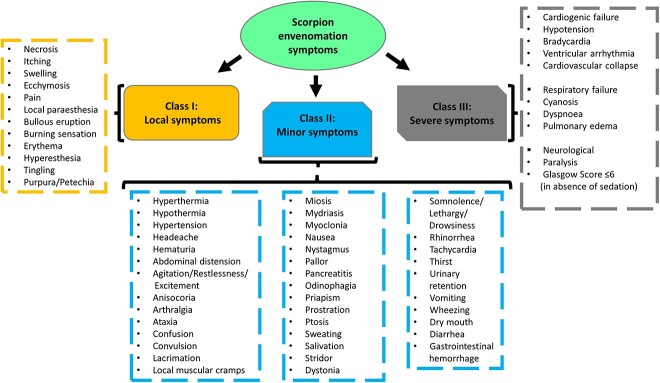
The classification of the signs and symptoms of scorpion sting envenomation.^9^^,^^70^

Although antivenom is the most effective treatment for scorpion envenoming, there are other alternatives to use including salicylates such as aspirin, which may reduce intense pain in both children and adults, recommended during class I (local effects) of the clinical symptoms in Fig. 1. At the site of the sting, nonsteroidal anti-inflammatory drugs such as indomethacin, diclofenac and application of lidocaine cream can also be used.^11^ Class II and III require antivenom, which is taken together with salicylates such as prazosin, which helps in cases involving hypertension or cardiac arrhythmia.^6^ In neuromuscular disorders, salicylates such as benzodiazepines can improve the effectiveness of the antivenom.^6^ Alternative substances that can be used are steroids and antihistamines, which reduce inflammation; calcium gluconate, which helps with easing muscle spasms; and sodium phenobarbital, which prevents lung edema and convulsions.^11^

In this review, we discuss the epidemiology of scorpion sting envenomation in Southern and Northern Africa. We also highlight the mechanism and clinical implication of scorpion envenomation. We further update the toxin components of Southern and Northern African scorpion venom and report current tools used in scorpion venomics and antivenoms in the treatment of scorpion envenomation in Southern and Northern Africa. Finally, we discuss the therapeutic applications of scorpion venom.

## Distribution of the northern and southern African scorpion family

In Southern Africa, four scorpion families have been identified which are Scorpionidae, Liochelidae, Bothriuridae, and Buthidae. The most scorpion stings of these families belong to the Buthidae and Scorpionidae.^12^ Some of the Buthidae, Scorpionidae, and Liochelidae scorpion families are also found in Northern Africa. Table 1 summarizes the most medically important scorpion species in the Buthidae family found in Southern and Northern Africa. The Buthidae scorpion family contains venom that can cause a life-threatening problem to humans and has attracted the attention of researchers.

**Table 1:** Summary of the most medically important Buthidae scorpions found in southern and northern Africa.

**Genus**	**Species**	**Distribution**	**References**
*Androctonus*	*amoreuxi*	Egypt, Morocco	^5^ ^,^ ^71^
	*australis*	Algeria, Egypt, Libya, Morocco, Tunisia	^5^ ^,^ ^7^
	*bicolor (aeneas)*	Algeria, Egypt, Libya, Morocco, Tunisia	^5^ ^,^ ^18^
	*crassicauda*	Algeria, Egypt, Morocco	^5^ ^,^ ^7^ ^,^ ^72^
	*liouvillei*	Morocco	^5^ ^,^ ^71^
	*mauretanicus*	Algeria, Morocco	^5^ ^,^ ^7^
*Buthus*	*lienhardi*	Morocco	^5^ ^,^ ^73^
	*malhommei*	Morocco	^5^ ^,^ ^72^
	*mardochei*	Morocco	^5^ ^,^ ^72^
	*paris*	Algeria, Morocco, Tunisia	^5^ ^,^ ^72^
	*tunetanus*	Algeria, Libya, Morocco, Tunisia	^5^ ^,^ ^72^
	*occitanus*	Morocco	^7^ ^,^ ^74^
*Parabuthus*	*granulatus*	Botswana, Limpopo (South Africa), North-West (South Africa), Northern Cape (South Africa), Western Cape (South Africa), Zimbabwe	^7^ ^,^ ^23^ ^,^ ^25^ ^,^ ^75^
	*capensis*	Northern Cape (South Africa), Western Cape (South Africa)	^25^ ^,^ ^75^
	*transvaalicus*	Botswana, Limpopo (South Africa), Gauteng (South Africa), North-West (South Africa), Mpumalanga (South Africa), Mozambique, Zimbabwe	^7^ ^,^ ^23^ ^,^ ^25^ ^,^ ^75^
	*schlechteri*	Northern Cape (South Africa), Western Cape (South Africa)	^25^ ^,^ ^75^
	*villosus*	South Africa, Namibia	^19^ ^,^ ^23^
	*raudus*	North-West (South Africa), Northern Cape (South Africa)	^25^ ^,^ ^75^
*Leiurus*	*quinquestriatus*	Egypt	^5^ ^,^ ^74^

### Buthidae family

The Buthidae family is the largest scorpion family, which comprises 86 currently recognized genera and over 900 valid species.^5^ It is widely distributed, occupying all the 6 faunal regions of the world. Buthidae is the main dangerous family of scorpions known to be the most medically important to humans all around the world.^5^^,^^13^^,^^14^ Identification of the scorpion species is difficult and only an expert can be able to identify the different species. The scorpion tail (telson) and the pincer size are used to differentiate the scorpion families. The Buthidae scorpion family have thick tails (telson) and smaller pincer sizes, while other scorpion families have thin tails.^12^ The genera found within the African Buthidae family are *Androctonus*, *Buthus*, and *Leiurus* found in Northern Africa, and *Parabuthus* found in Southern Africa (Table 1).^15^

### *Androctonus* genus

The *Androctonus* (fat-tailed scorpions) genera are typically found in diverse locations ranging from elevated mountain regions to deserts and in human settlements, thus elevating the risk of envenomation, which is supported by their frequent incrimination for severe stings.^16^*Androctonus* has 22 species, of which 6 are distributed in North African countries such as Algeria, Egypt, Libya, Morocco, and Tunisia.^17^ The scorpions found in these regions are *Androctonus amoreuxi, Androctonus australis, Androctonus bicolor (aeneas*), *Androctonus crassicauda*, *Androctonus liouvillei*, and *Androctonus mauretanicus* (Table 1). Interestingly, all these *Androctonus* genera are present in Morrocco. Here, the *Androctonus* species appears to be responsible for many medically significant scorpion stings, with an estimated number of about 50,000 cases reported to the Moroccan Poison Information Center (PIC) yearly.^18^ However, only a minority of scorpion species are known to cause medically significant envenoming in humans. *A. australis* in Algeria is reported to cause about 70% medically significant stings, while *A. crassicauda* is considered the deadliest scorpion. This scorpion is blackish brown in body color, and it has a body size of over 100 mm in length (Table 1).^5^^,^^18^

### *Leiurus* genus

The *Leiurus* genera are comprised of 16 species, but it was reported that all the species were represented by only *Leiurus quinquestriatus* (deathstalker scorpion) (Table 1), which is a well-known, recognized and feared dangerous species of the Sahara (particularly in Northern Niger and Southern Egypt).^19^ It is orangish-yellow in body color, and its length ranges from 90–110 mm. Members of *Leiurus* genera can be found across Africa and the Middle East, in desert regions as they have evolved to thrive in these dry and hot climates and are also known to be medically important species.^5^^,^^20^^,^^21^

### *Buthus* genus

*Buthus* is the fourth largest genera of the Buthidae family, with about 52 identified species, and 6 of these species (*B. paris*, *B. tunetanus*, *B. malhommei*, *B. occitanus*, *B. lienhardi*, and *B. mardochei*) are medically important (Table 1). In this genera, *B. occitanus* is considered the cause of severe scorpion stings in Morocco. It is also called Languedoc yellow scorpion which is yellow in body color with darker tergites and prosoma. The average body size ranges from 45–80 mm in length.^5^^,^^22^ Once again, all the scorpions from the *Buthus* genus were present in Morocco (Table 1).

### *Parabuthus* genus

The *Parabuthus* genera are comprised of 22 species, which are identified in Southern Africa. This genus has multiple medically important species and is the only genus to have both the world’s biggest scorpions and the only diurnal scorpion (*P. villosus*), found in Southern Africa.^19^^,^^23^ Members of this genus share the physical feature of thick tails, hence why they are dubbed thick-tailed scorpions.^23^^,^^24^*P. granulatus* and *P. transvaalicus* are responsible for severe envenomation in Southern Africa. Their body size ranges from 60–150 mm in length, and *P. transvaalicus* is differentiated from *P. granulatus* by body color, where the former is overall black while the latter is light to dark brown in color. *P. transvaalicus* is dominant in Zimbabwe and is known to have the highest sting incidences per 100,000 inhabitants, while *P. granulatus* is the most dangerous scorpion species in South Africa which is responsible for the majority of scorpion envenomation and deaths, with the most calls originating from the Western Cape and Gauteng (Table 1).^8^^,^^25^

## Mechanism and clinical manifestations of scorpion envenomation

There are about less than 20% of cases of severe scorpion envenoming that cause a life-threatening issue. The following factors determine the severity of the scorpion stings: the species involved, size, age, nutrition, climatic condition of the habitat, delay in taking care, and amount of venom injected. High severity is typically seen in children and in vascularized areas of the body, such as the neck, head, or trunk.^6^^,^^26^ The act of injecting venom into a victim’s body by stinging, biting, spraying, or spitting is known as envenomation.^27^ The venom glands of the scorpions are located at the tail (telson), which is used to inject the venom into a victim’s body.^11^ Scorpion venom is made up of different components which play a role in its toxicity and potency, and other factors such as age, sex, and geographical distribution also aid in some of the chemical components being more prominent in some scorpions than others.^14^ Numerous toxins found in scorpion venoms have been identified and most are small peptide toxins that target the ion channels found in both insects and mammals.^4^ Scorpion α-toxins are considered to have the greatest medical consequences and are made up of 61 to 76 polypeptides that bind to a specific site on the voltage-gated sodium channel in mammals. After the binding of toxin to a site, it prevents the inactivation of the channels, prolonging depolarization and ultimately causing neuronal excitation. Other toxins that seem to be less important in human envenomation act on potassium and calcium channels.^4^

The sympathetic and parasympathetic autonomic centers are stimulated by the excitation of neurons, which results in autonomic excitation. In addition, the release of catecholamines epinephrine, norepinephrine, and vasoactive peptide hormones such as neuropeptide Y and endothelin-1 are caused by scorpion α-toxins.^4^ The parasympathetic effects are less severe when compared to sympathetic effects and occur early after the sting. Severe systemic effects such as cardiogenic shock, myocardial injury, and pulmonary edema are caused by the sympathetic excitation and release of catecholamine.^4^

The venom injected into a human can cause various effects ranging from local pain and inflammation to tissue damage depending on the type of scorpion species that has stung a person in a particular region. According to Vaucel et al., scorpion envenoming consists of three (3) classes of symptoms which are class I (local symptoms), class II (minor symptoms), and class III (severe symptoms) (Fig. 1).^9^ Severe localized pain is the first symptom as the venom penetrates through the body and, in children, is a valuable warning signal. In about 95% of cases of envenoming, it was found that pain is present and is associated with local manifestations such as itchiness, edema, and erythema, which fall under class I (Fig. 1).^6^^,^^26^

The dangerous scorpion species cause systemic manifestations that result from the action of neurotoxins on sodium channels, causing a release of neurotransmitters. These systemic manifestations are indicated by the signs and symptoms involving autonomic nervous systems (ANS), central venous systems (CNS), and can include respiratory and heart failure, potentially leading to death.^26^

The ANS excitation is characterized by both parasympathetic and sympathetic responses. The symptoms of cholinergic effects, which is a parasympathetic response, include hypotension, diarrhea, hypersalivation, vomiting, priapism, lacrimation, increased respiratory secretions, miosis, and profuse diaphoresis. Most of the parasympathetic symptoms belong to both class II and class III and tend to occur early (Fig. 1). Symptoms of adrenergic effects, which is a sympathetic response due to the release of catecholamines, include hyperglycemia, hypertension, agitation, tachycardia, restlessness, mydriasis, and hyperthermia, and they are responsible for severe envenomation.^4^^,^^28^ Adequate reporting of scorpion stings and associated symptoms can be helpful in understanding the mechanism of action of the venom and better classification of the clinical manifestations.

## Scorpion venom composition

**Fig 2:**
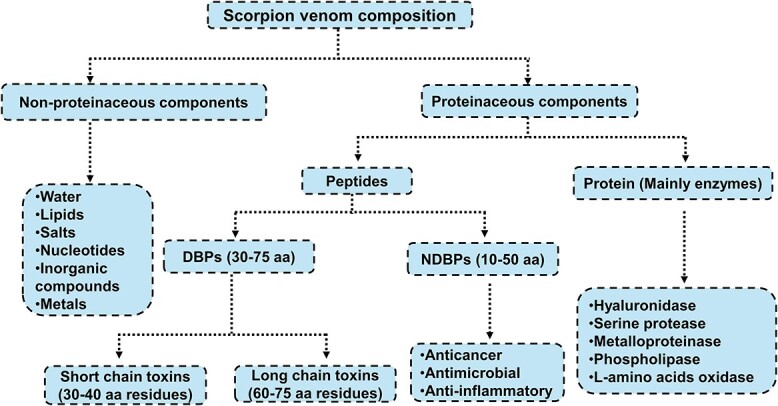
A summary of the components found in scorpion venom. Amino acid (aa), disulfide-bridged peptides (DBPs), and non-disulfide-bridged peptides (NDBPs).^31^

According to Tobassum et al (2018), scorpion venom comprises different complex mixtures of lipids, peptides, enzymes, free amino acids, nucleotides, amines, inorganic salts, mucoproteins, heterocyclic components, and several other unknown substances (Fig. 2).^29^ As mentioned above, the early symptoms observed in envenomed people are due to the scorpion venom being very toxic with a very fast diffusion and action. The use of biochemical techniques has revealed the complex composition of the scorpion venom with toxic and non-toxic fractions. The mixture of enzymes such as hyaluronidases and phospholipases, mucopolysaccharides, protease inhibitors, and bioamines such as histamine and serotonin are classified as non-toxic fractions (Fig. 2).^26^ The toxic fraction is highly studied due to the clinical relevance of neurotoxins, which bind to the ion channels of excitable cells and are different in their degree of toxicity and affinity to specific targets of animal species. These neurotoxins are responsible for the toxic manifestations observed in patients. Again, it should be mentioned that the composition of scorpion venom, which determines its toxicity, depends on age, sex, diet, and geographical origins, amongst other factors.^26^

Scorpion venom toxins have an immense impact on mammals, including humans. The toxins that block sodium channels are the most significant, which are categorized as the α-toxins that cause the delay of inactivation of voltage-gated sodium channels and β-toxins that cause the opening of the channels at high negative potentials.^30^ In low dosages of the venom, the α-toxins cause a significant depolarization of the cell membrane, which is followed by a decrease in excitability but prolongs the potential action of excitable cells at higher doses, which causes paralysis and cardiac arrhythmia.^6^ The β-toxins cause myoclonic or spastic muscular responses. The other scorpion venom toxins affect the membrane ion channels, such as potassium, calcium, and chlorine. These toxins can display actions that lead to clinical manifestations, but they seem to have a less important role in human envenomation. All these toxins combined can have an impact on clinical symptoms which may result in severe complications or paradoxical syndromes. For example, β -toxins alone cause mild clinical effects; however, when combined with other toxins, the interaction between the toxins causes more severe clinal effects.^6^^,^^30^

Other components found in scorpion venom are the non-disulfide-bridged peptides (NDBPs) and disulfide-bridged peptides (DBPs). The NDBP components are small amino acids made up of 13 to 50 amino acid residues and are considered to be the major component of peptides in scorpion venom. Mass-fingerprint analyses of scorpion venom have shown that low molecular weight peptides present are more than a third of all identified peptides. As shown in Fig. 2, the DBPs are classified into two categories which are the short-chain toxins which are made up of 10 to 40 amino acid residues and the long-chain toxins which are made up of 60 to 75 amino acid residues stabilized by 3 or 4 disulfide bonds, and affect Na^+^, K^+^, Ca^2+,^ and Cl^−^ channels.^30^^,^^31^

The enzymes such as metalloproteases, hyaluronidases, and phospholipases found in scorpion venom aid in the toxicity of the venom. For instance, hyaluronidase is known as a spreading factor of the toxins.^11^^,^^29^ This is due to the hyaluronidase disrupting the extracellular matrix and connective tissues surrounding the blood vessels at the sting point, which results in the toxins spreading faster to their target. On the other hand, phospholipases are potent hemolytic agents; they disrupt the cell membrane by hydrolyzing the phospholipids, which results in tissue necrosis and hemorrhages.^11^^,^^29^ The protease enzymes play a key role in the activation of venom toxin precursors through post-translational modifications. These enzymes are also known to inhibit platelet aggregation, activation of the complement system, and modulation of cytokine production. Altogether, these effects cause the spreading of the venom toxins through matrix protein degradation. Neutralizing these enzymes could be considered the first-aid treatment for scorpion envenoming.^11^^,^^29^

## Tools for the analysis of scorpion venom

With emerging “omic” technologies, such as proteomic and transcriptomic analyses, the biological roles of scorpion venom and their applications in research can be improved. Proteomics is the study and evaluation of the proteome within a given sample (e.g. scorpion venom), including its composition and function at a given point in time.^32^ Venomics is a branch of science that specifically studies the proteins and peptides associated with venom through proteomic, transcriptomic, and genomic analyses.^33^^,^^34^ Other fields of study, including toxicovenomics and antivenomics, also employ these emerging “omics” technologies to study how venom toxins could affect biological systems and antivenom efficiency.^33^ The proteomic or venomic approach of analysing toxin composition can be completed directly by utilizing the strategy centred around the proteins or indirectly by high-throughput transcriptomics of the venom glands and computational analysis.^35^ The advantages of scorpion venom proteomic analysis include the elucidation of venom composition, the increased understanding of molecular mechanisms of venom toxicity, the further understanding of protein–protein interactions by imaging the intra- and interspecific structures of the proteins, and lastly, the addition of knowledge to potential therapeutic and diagnostic agents amongst others.^32^^,^^36–38^

Traditional methods of conducting proteomic studies include the use of gel electrophoresis in one and two dimensions (1DE and 2DE), reverse-phase high-performance liquid chromatography (RP-HPLC), and mass spectrometry (MS) methods such as matrix-assisted laser desorption/ionization time-of-flight (MALDI-TOF).^36^^,^^39^ All the above methods are used to create venom fractionation profiles and reveal the quantity of molecular toxins.^35^

The proteomic workflow methods can either be bottom-up (peptide-based proteomics) or top-down (“shotgun” proteomics).^32^^,^^40^ According to Al-Amrani et al. and Mouchbahani-Constance and Sharif-Naeini, the bottom-up method involves the protein isolation and excision of spots on a 2DE gel, followed by the digestion of those proteins. After digestion, the proteins are fractionated using liquid chromatography (LC), and subsequent MS analysis is completed. The resulting fragmentation pattern is analyzed, and the fragments can be identified by comparing them to protein sequence libraries.^32^^,^^40^^,^^41^ There are limitations to the bottom-up method because the digestion process cleaves the proteins into smaller peptides before MS analysis, resulting in the loss of the full-length protein structure, along with possible post-translation modifications (PTMs).^32^^,^^41^ Hence, this loss results in a low coverage percentage of the protein sequence.^32^ In contrast, the top-down approach utilizes MS to study the whole (undigested and non-fragmented) proteome.^32^^,^^34^

Gel electrophoresis is another powerful tool used in proteomics that can be used alone or in tandem with liquid chromatography. This (gel electrophoresis) technique separates analytes (e.g. venom proteins and peptides) according to their molecular weights, shapes, and electric charge.^42^ Protein characterization can be completed with the simple denaturing 1DE, where the proteins are denatured using a detergent such as sodium dodecyl sulphate (SDS) and separated according to their molecular weight.^32^^,^^42^ On the other hand, 2DE is considered a superior method to 1DE as it separates the proteins according to their isoelectric point (pI) in one dimension and molecular weight in the second.^43^^,^^44^ Lastly, gel staining can indicate the presence of varying PTMs.^43^

RP-HPLC is a high-resolution separation method for venom proteins and peptides. The venom components are typically separated on a C_18_ column, thus resulting in analytes eluting from the column in order of polar to non-polar.^41^ RP-HPLC is advantageous because it allows for the fractionation of protein isoforms with high precision.^41^^,^^44^ Following fractionation, MALDI-TOF/MS can provide information about the proteins following a three-step process, including analyte ionization, analysis and detection.^41^ This resulting MS fragmentation spectra indicate the mass-to-charge ratio of fragments, which can be compared to existing protein libraries to identify the proteins.^41^^,^^42^ Lastly, MALDI-TOF generates spectra illuminating proteins’ structure, mass, chemical compositions, and other biological components.^41^ To date, in the venom of about 27 scorpion species, an estimated number of about 5,300 molecular masses have been identified using spectrometric analyses, and it was found that 70.6% are from the Buthidae family. For example, a study done by Abdel-Rahman et al. used a proteomics or transcriptomics approach to study the differences among the species belonging to the various scorpion families such as Buthidae, Urodacidae, Scorpionidae, etc. The data obtained revealed that environmental/biological factors such as area, age, climate, and sex play a role in the venom components of different scorpion species. So far, the venom components of Moroccan *B. occitanus* have been identified by using proteomics approaches. In Southern and Northern Africa, there is still a gap in the knowledge of the scorpion venom composition that can cause a life-threatening problem for humans. Understanding and revealing the compositions of these scorpion species will aid in the production of board-spectrum antivenom.^45^

## Production and distribution of antivenom

**Fig. 3:**
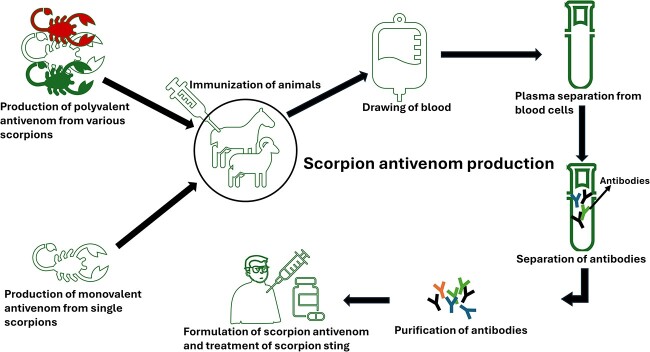
The production of polyvalent/monovalent scorpion antivenom. Animals are immunized with scorpion venom over a period until the animal has produced antibodies that can neutralize the venom, after which the blood is then drawn. Following the processing and purification of antibodies, an antivenom vial is produced, which is either in a powder/liquid and can be used to treat scorpion stings.^11^

Antivenom is the mosteffective treatment against scorpion envenomation. It can be defined as a biological product that is developed following the vaccine principle. There are multiple methods available to produce antivenom. However, the most common approach involves the purification of polyclonal antibodies from immunized horses or sheep.^11^ The process involves exposure to increasing concentrations of one or multiple scorpion species’ venom until the horse or sheep has developed venom-specific antibodies.^46^ The blood is then drawn, and the plasma is isolated and purified to extract the antibodies utilizing techniques like affinity chromatography, which is highly specific. The concentration of the antibodies is closely monitored prior to the production of the antivenom, which can then be used for the treatment of severe envenomation (Fig. 3).^11^ The same procedure is used to produce snake antivenom.^47^ The resulting antivenom can either be specific to the venom of one species (monovalent) or against numerous species (polyvalent) (Fig. 3).^46^

The majority of in-market scorpion antivenoms are typically polyvalent as they have a more potent effect than monovalent antivenoms and can be used for the treatment of numerous scorpion species stings. It is for this reason and the steady rise of scorpion envenomation and expected increases in the future that the WHO has urged scientists to continue developing polyvalent antivenoms utilizing highly venomous scorpion species to try and combat cases of severe envenoming, as antivenoms are acknowledged as the most effective treatment.^48^

Table 2 lists antivenoms commercially available in Southern and Northern African countries. There are currently 20 in-market scorpion antivenoms, of which 19 are for human use and 1 for animal use.^49^ In Northern Africa, there are about 7 antivenoms available to treat victims of scorpion stings, and in Southern Africa, only 1 scorpion antivenom is currently available (Table 2). While monovalent antiscopionique antivenom was able to neutralize venom from *A. australis* produced by the Institut Pasteur d’Alegrie, Algeria, bivalent antivenom produced by Institut Pasteur de Tunis, Tunisia neutralized *A. australis* and *B. occitanus* venoms. Additionally, polyvalent antivenom produced by Institut Pasteur du Maroc, Morocco was able to neutralize venoms from *B. occinatus, A. mauritanicus.* However, there has been a decrease in antivenom production as it is a costly, laborious, and time-consuming process yielding minute volumes of venom during milking and collection, thus making it insufficient for antisera production as this requires large volumes.^49^ Potent et al. stated several reasons as to why the global output of antivenom was insufficient listing a limited number of producers in a particular country as one.^50^ It should also be noted that antivenom production is location-specific, with many manufacturers focusing on species that are native to those areas, leaving potentially invasive and less venomous species without a method of treatment.^51^^,^^52^ Furthermore, antivenom is not easily accessible as many people in high-risk and impoverished tropical and sub-tropical regions do not have access to proper healthcare facilities, thus leading to severe envenomation and possibly death.^7^^,^^47^

**Table 2:** Scorpion antivenom currently available for use in Southern and Northern Africa.^5^^,^^49^

**Antivenom name**	**Manufacturer and Location**	**Species neutralized**
SAIMR scorpion antivenom	South African Vaccine Producers, SA	*P. transvaalicus*
Bivalent scorpion antivenom	Institut Pasteur de Tunis, Tunisia	*A. australis, B. occinatus*
Anti-scorpionic sera		*A. australis, A. bicolor, B. occinatus, L. quinquestriatus*
Polyvalent scorpion antivenom	Institut Pasteur du Maroc, Morocco	*B. occinatus, A. mauritanicus*
Anti-scorpionique (Monovelent)	Institut Pasteur d’Alegrie, Algeria	*A. australis*
Purified polyvalent anti-scorpion serum	Egyptian Organization for Biological Products and Vaccines (VACSERA), Egypt	*A. amoreuxi, A. australis, A. bicolor, A. crassicauda, B. occitanus, L. quinquestriatus, Scorpio maurus*
Scorpifav	Sanofi Pasteur, NA	*A. Australis, L. quinquestriatus, Hemiscorpius lepturus, Hottentotta saulcyi, Hottentotta Schach, Mesobuthus eupeus, Odontobuthus doriae*
Scorpion antivenom Twyford	Twyford Pharmaceuticals, NA	*A. australis, B. occitanus, L. quinquestriatus*

It is also important to note that the antivenoms currently in-market are imperfect as they may result in adverse reactions being experienced due to the differences in how the venom composition of various species has been modified based on their age, sex, geographical distribution, and diet.^26^ Therefore, the scorpion envenoming treatment with antivenoms is still under debate. This is due to a study by Abroug et al. in Tunisia, where about 875 patients were treated with the bivalent antivenom product of Institut Pasteur de Tunis, which did not show any benefits. It was assumed that the envenoming was caused by *A. australis* and *B. occitanus*.^53^ There are, however, some cases that have indicated the effectiveness of some antivenoms. In addition, antivenoms derived from equine hosts may cause acute hypersensitive reactions or anaphylaxis; however, increasing the infusion duration may mitigate this.^49^Although the use of scorpion antivenom is encouraged due to several examples reported in this review (Table 2), it is therefore imperative to further our knowledge of the variations in venom compositions between species and the classes of envenomation symptoms caused by scorpion stings. This will enable the development and distribution of more effective antivenoms to high-risk and impoverished regions in Southern and Northern Africa.^29^^,^^54^^,^^55^

## Therapeutic applications of southern and northern Africa scorpion venoms

Scorpion venom is known for its deadly effects on organisms, cells, and tissues. Since ancient cultures, scorpions have been used in traditional medicine for the treatment of many diseases in Africa, Asia, and India.^29^^,^^56^ It was reported that scorpion venom is a rich source of biologically active peptides with therapeutic properties. Bioactive compounds can be found in natural resources, including fungi, plants, and bacteria, but biologically active peptides found in venomous species have shown a variable function, high selectivity, and specificity to human target cells.^56^ Scorpion venom has been studied for more than two decades; with the help of novel proteomics methodologies, different components found within the scorpion venom have been identified and characterized. It is known that scorpion venom is made up of complex mixtures, as previously discussed. Some of the toxins and peptides found within the scorpion venom have the ability to act on protein targets with potency and selectively, exhibiting pharmacological effects relevant to human disorders and diseases.^29^^,^^57^ These toxins and peptides have displayed therapeutic properties that make them suitable candidates for drug development for the treatment of many diseases. As previously mentioned, scorpion toxins have an important effect on excitable cells due to their targeting ion channels, including calcium, potassium, chloride, and sodium. Therefore, these toxins can be considered when designing drugs for cardiovascular diseases.^56^ According to Ortiz et al. NDBPs found within the scorpion venom show multifunctional activities that can be used to treat various diseases. These activities include anticancer, antiviral, antifungal, antiparasitic, antibacterial, autoimmune, anti-inflammatory, and cardiovascular disease.^30^ In Northern and Southern Africa, some scorpions have shown some therapeutic activities such as anticancer, cardiovascular diseases, autoimmune, antimicrobial, and analgesic activity.^58^ The discovery of the NDBP candidates has brought interest to scientists as these peptides show different therapeutic activities and are promising candidates for therapeutic drugs.^30^

For instance, *L. quinquestriatus* found in Northern Africa was found to have chlorotoxin (ClTx), a component that interacts with the chloride channels. This toxin inhibits the chloride invading the glioma cells by binding to the matrix metalloproteinase II (MMP-II) expressed by the glioma cells. The MMP-II breaks down and remodels the extracellular matrix, which aids in the normal tumor cells being able to penetrate through the tissue barriers, while the ClTx inhibits the enzymatic activity of MMP-II and reduces its expression (Table 3).^11^^,^^59^ ClTx from L. *quinquestriatus quinquestriatus* venom and its synthetic derivatives (TM-601) are undergoing clinical trials.^60–62^*A. australis* found in Tunisia was found to contain a chlorotoxin-like peptide (AaCtx) that shares about 70% similar amino acid sequence with chlorotoxin from *L. quinquestriatus*. This AaCtx was found to inhibit the migration and invasion of the human glioma cells (U87).^63^

**Table 3:** A summary of some scorpion species within southern and northern Africa found to have therapeutic activities.

**Species**	**Locations**	**Crude venom/component**	**Therapeutic role**	**References**
*L. quinquestriatus*	Northern Africa	Chlorotoxin (ClTx),	Anticancer activity	^11^ ^,^ ^76^
*A. crassicauda*	Northern Africa	Crude venom	Anticancer activity	^11^ ^,^ ^65^ ^,^ ^76^
*A. australis*	Egypt	Crude venom	Anticancer activity	^29^ ^,^ ^77^
	Tunisia	Chlorotoxin (AaCtx)	Antimicrobial, Anticancer activity	^63^
*Allopauropus mauretanicus*	Morocco	Kaliotoxin (KTX), Mauriporin	Anticancer activity, Autoimmune diseases	^29^ ^,^ ^66^
*A. bicolor* (a*eneas*)	North Africa	AaeAP1 & AaeAP2 *(Androctonus aeneas* antimicrobial peptide)	Anticancer activity, Antimicrobial activity	^67^
*A. amoreuxi*	Egypt	Crude venom	Anticancer activity, Antimicrobial activity	^3^ ^,^ ^64^ ^,^ ^76^
*B. occitanus tunetanus*	Tunisia	RK1	Anticancer activity	^66^
*P. schlechteri*	Southern Africa	Parabutoporin	Antimicrobial	^69^
*Opistophtalmus carinatus*	South Africa	Opistoporin 1 and Opistoporin 2	Antifungal activity, Antimicrobial activity	^69^

According to Salem et al. *A. crassicauda* and *A. bicolor* have shown potential anticancer effects on colorectal and breast cancer cell lines through the decreasing formation of cancer cells motility (Table 3).^64^ Mauriporin is a peptide found in *A. mauritanicus,* which also showed antiproliferation activity against prostate cancer cell lines. It was found that Mauriporin does not induce cell death through the apoptotic pathway but possibly through the necrotic mode of cell death.^65^ RK1 was the first peptide found in *B. occitanus tunetanus*. It was found to inhibit the proliferation of glioblastoma (U87) cells, and it was also found that more than 90% of the proliferation of melanoma cells (IGR39) was inhibited.^66^

*A. bicolor* from North Africa was found to contain two antimicrobial peptides (AaeAP1a and AaeAP2a). They have shown growth-inhibitory activities against Gram-positive bacteria (*Staphylococcus aureus*) and yeast (*Candida albicans*). These peptides have also shown activity of inhibiting the proliferation of four human cancer cells (human prostate carcinoma cell line (PC-3), human lung adenocarcinoma cell line (NCI-H460), human breast carcinoma cell line, MDA-MB-435s, non-tumourigenic mammary gland cell line, and MCF-7) in a dose-dependent manner (Table 3).^67^*A. mauretanicus* was found to contain a kaliotoxin (KTX), a component that blocks the potassium (K^+^) channel, which is considered to be a suitable pharmacological target for immunosuppressive therapy.^29^^,^^68^*P. schlechteri* contained Parabutoporin, and *O. carinatus* contained Opistoporin 1 and 2, which were found to inhibit the growth of Gram-negative bacteria. Opstoporin I also inhibited the growth of *Neurospora crassa*, *Fusarium culmorum* fungus, and *Saccharomyces cerevisiae* yeast.^69^

## Conclusion and perspectives

Although scorpion stings can pose a medical emergency in many Southern and Northern African regions, it is important to note that not all scorpion stings are medically significant. However, when they do occur, the severity of the stings tends to be more pronounced in children and the elderly. Differences in the geographical distribution of scorpions in Southern and Northern Africa result in varying venom toxicity and pharmacological effects. These differences often lead to variations in both local and severe systemic symptoms. In-depth analysis of the venom proteome is required to determine the relationship between the venom composition and the severity of the sting of the various species of scorpions. This will help in the production of a broad-spectrum and more effective antivenom to treat scorpion envenoming within Southern and Northern Africa. Although there are several commercially available antivenom useful in treating and managing scorpion envenoming in Southern and Northern Africa, there are alternative treatments that can complement its use. The combination of immunotherapy plus symptomatic treatment is still often required in many cases. These therapeutic approaches potentiate each other and provide a good response to most common situations encountered in tropical and subtropical regions. Training in these combined treatment protocols should be provided to health professionals in Southern and Northern Africa regardless of the severity of scorpion envenomation. Additionally, the use of omics technologies, such as proteomics, transcriptomics and genomics are instrumental in the study of scorpion venom composition which is key in elucidating venom toxin variations. Scorpion venom toxins from these two regions have shown promise as prospective therapeutics for the treatment of diseases, including cancer. More research is needed to upscale the progression of these venom peptide toxins beyond pre-clinical studies and eventual drug production.

## References

[1] Kumar R . An update on epidemiology and management practices of scorpion envenomation in India. J Family Med Prim Care. 2022:11(9):4932–4935. 36505581 10.4103/jfmpc.jfmpc_2300_21 PMC9731072

[2] Petricevich VL . Scorpion venom and the inflammatory response. Mediat Inflamm. 2010:2010:903295. 10.1155/2010/903295 PMC2838227 20300540

[3] Peter Muiruri K , Zhong J, Yao B, Lai R, Luo L. Bioactive peptides from scorpion venoms: therapeutic scaffolds and pharmacological tools. Chin J Nat Med. 2023:21(1):19–35. 36641229 10.1016/S1875-5364(23)60382-6

[4] Isbister GK , Bawaskar HS. Scorpion envenomation. N Engl J Med. 2014:371(5):457–463. 25075837 10.1056/NEJMra1401108

[5] Jenkins T , Ahmadi S, Bittenbinder M, Stewart T, Akgun D, Hale M, Nasrabadi NN, Wolff D, Vonk F, Kool J, et al. Terrestrial venomous animals, the envenomings they cause, and treatment perspectives in the Middle East and North Africa. PLoS Negl Trop Dis. 2021:15(12):e0009880. 34855751 10.1371/journal.pntd.0009880 PMC8638997

[6] Chippaux JP . Emerging options for the management of scorpion stings. Drug Des Devel Ther. 2012:6:165–173. 10.2147/DDDT.S24754 PMC3401053 22826633

[7] Chippaux JP , Goyffon M. Epidemiology of scorpionism: a global appraisal. Acta Trop. 2008:107(2):71–79. 18579104 10.1016/j.actatropica.2008.05.021

[8] Marks CJ , Muller GJ, Sachno D, Reuter H, Wium CA, Du Plessis CE, Van Hoving DJ. The epidemiology and severity of scorpion envenoming in South Africa as managed by the Tygerberg poisons information Centre over a 10 year period. Afr J Emerg Med. 2019:9(1):21–24. 30873347 10.1016/j.afjem.2018.12.003 PMC6399994

[9] Vaucel JA , Larréché S, Paradis C, Courtois A, Pujo JM, Elenga N, Résière D, Caré W, de Haro L, Gallart JC, et al. French Scorpionism (mainland and oversea territories): narrative review of scorpion species, scorpion venom, and envenoming management. Toxins. 2022:14(10):719. 36287987 10.3390/toxins14100719 PMC9611377

[10] World Health Organization . Report of the eleventh meeting of the WHO strategic and technical advisory group for neglected tropical diseases. France: World Health Organization; 2018 pp. 1–28 Retrieved from https://www.who.int/publications/m/item/eleventhreport-of-the-strategic-andtechnical-advisory-group-forneglected-tropical-diseases-(stag-ntds).

[11] Ahmadi S , Knerr JM, Argemi L, Bordon KCF, Pucca MB, Cerni FA, Arantes EC, Çalişkan F, Laustsen AH. Scorpion venom: detriments and benefits. Biomedicines. 2020:8(5):118. 32408604 10.3390/biomedicines8050118 PMC7277529

[12] Müller GJ , Modler H, Wium CA, Veale DJH. Scorpion sting in southern Africa: diagnosis and management. Contin Med Educ. 2012:30(10):356–361.

[13] Goyffon M , Tournier JN. Scorpions: a presentation. Toxins. 2014:6(7):2137–2148. 25133517 10.3390/toxins6072137 PMC4113747

[14] Durden LA, Mullen GR. Introduction. Medical and Veterinary. (3rd eds) Chapter 1, 2019:1–16. 10.1016/B978-0-12-814043-7.00001-7.

[15] Mullen GR, Sissom WD. Scorpions (Scorpiones). In: Mullen GR, Durden LA. Medical and veterinary entomology. United States: Elsevier, (3rd eds) chapter 23, 2019:489–504. 10.1016/B978-0-12-814043-7.00023-6.

[16] El Hidan MA , Touloun O, Bouazza A, Laaradia MA, Boumezzough A. Androctonus genus species in arid regions: ecological niche models, geographical distributions, and envenomation risk. Vet World. 2018:11(3):286–292. 29657418 10.14202/vetworld.2018.286-292 PMC5891841

[17] Coelho P , Sousa P, Harris DJ, Van der Meijden A. Deep intraspecific divergences in the medically relevant fat-tailed scorpions (Androctonus, Scorpiones). Acta Trop. 2014:134(1):43–51. 24524948 10.1016/j.actatropica.2014.02.002

[18] White J. Overview of scorpion envenoming. In: Brent J, Burkhart K, Dargan P, Hatten B, Megarbane B, Palmer R. (eds) Critical Care Toxicology. Springer, Cham. 2016:1–15. 10.1007/978-3-319-20790-2_147-1.

[19] Goyffon M , Dabo A, Coulibaly SK, Togo G, Chippaux JP. Dangerous scorpion fauna of Mali. J Venom Anim Toxins Incl Trop Dis. 2012:18(4):361–368.

[20] Alqahtani AR , Badry A. Genetic diversity among different species of the genus Leiurus (Scorpiones: Buthidae) in Saudi Arabia and the Middle East. Saudi J Biol Sci. 2020:27(12):3348–3353. 33304141 10.1016/j.sjbs.2020.08.048 PMC7715040

[21] Lourenço WR . Scorpion incidents, misidentification cases and possible implications for the final interpretation of results. J Venom Anim Toxins Incl Trop Dis. 2016:22(1):1–25. 10.1186/s40409-016-0075-6 PMC4938980 27398081

[22] Colombo M . On Fabre’s traces: an important contributor to the knowledge of *Buthus occitanus* (Amoreux, 1789). Euscorpius. 2011:2011(117):1–10.

[23] Debont T , Swerts A, Van Der Walt JJ, Müller GJ, Verdonck F, Daenens P, Tytgat J. Comparison and characterization of the venoms of three *Parabuthus* scorpion species occurring in southern Africa. Toxicon. 1998:36(2):341–352. 9620581 10.1016/s0041-0101(97)00099-8

[24] Prendini L , Esposito LA. A reanalysis of *Parabuthus* (Scorpiones: Buthidae) phylogeny with descriptions of two new *Parabuthus* species endemic to the central Namib gravel plains, Namibia. Zool J Linnean Soc. 2010:159(3):673–710.

[25] Bergman NJ . Scorpion sting in Zimbabwe. S Afr Med J. 1998:87(2):163–167. 9107222

[26] Laraba-Djebari F, Adi-Bessalem S, Hammoudi-Triki D. Scorpion venoms: pathogenesis and biotherapies. In: Gopalakrishnakone P, Possani LD, Schwartz EF, Rodríguez de la Vega. Scorpion Venoms. Netherlands, Dordrecht, Springer, 2014:63–85.

[27] World Health Organization . Snakebite envenoming. Geneva, Switzerland: WHO; 2019 [Accessed 9 Mar. 2023]. Available at: https://www.who.int/news-room/fact-sheets/detail/snakebite-envenoming

[28] Agrawal A , Kumar A, Consul S, Yadav A. Scorpion bite, a sting to the heart! Indian J Crit Care Med. 2015:19(4):233–236. 25878433 10.4103/0972-5229.154570 PMC4397632

[29] Tobassum S , Tahir HM, Arshad M, Zahid MT, Ali S, Ahsan MM. Nature and applications of scorpion venom: an overview. Toxin Rev. 2020:39(3):214–225.

[30] Ortiz E , Gurrola GB, Schwartz EF, Possani LD. Scorpion venom components as potential candidates for drug development. Toxicon. 2015:93:125–135. 25432067 10.1016/j.toxicon.2014.11.233 PMC7130864

[31] Abdel-Rahman MA , Quintero-Hernández V, Possani LD. Scorpion venom gland transcriptomics and proteomics: An overview. In: Gopalakrishnakone P, Calvete JJ, editors. Venom genomics and proteomics. Netherlands, Dordrecht: Springer; 2016. pp. 105–124 10.1007/978-94-007-6416-3_29

[32] Al-Amrani S , AL-Jabri Z, Al-Zaabi A, Alshekaili J, Al-Khabori M. Proteomics: concepts and applications in human medicine. World J Biol Chem. 2021:12(5):57–69. 34630910 10.4331/wjbc.v12.i5.57 PMC8473418

[33] Slagboom J , Kaal C, Arrahman A, Vonk FJ, Somsen GW, Calvete JJ, Wüster W, Kool J. Analytical strategies in venomics. Microchem J. 2022:175:107187.

[34] Wilson D , Daly N. Venomics: a mini-review. High Throughput. 2018:7(3):19. 30041430 10.3390/ht7030019 PMC6164461

[35] Warrell DA , Gutiérrez JM, Calvete JJ, Williams D. New approaches & technologies of venomics to meet the challenge of human envenoming by snakebites in India. Indian J Med Res. 2013:138(1):38–59. 24056555 PMC3767246

[36] Abdel-Rahman MA , Quintero-Hernandez V, Possani LD. Venom proteomic and venomous glands transcriptomic analysis of the Egyptian scorpion *Scorpio maurus palmatus* (Arachnida: Scorpionidae). Toxicon. 2013:74:193–207. 23998939 10.1016/j.toxicon.2013.08.064

[37] Dias MH , Kitano ES, Zelanis A, Iwai LK. Proteomics and drug discovery in cancer. Drug Discov Today. 2016:21(2):264–277. 26484434 10.1016/j.drudis.2015.10.004

[38] Amiri-Dashatan N , Koushki M, Abbaszadeh H-A, Rostami-Nejad M, Rezaei-Tavirani M. Proteomics applications in health: biomarker and drug discovery and food industry. Iran J Pharm Res. 2018:17(4):1523–1536. 30568709 PMC6269565

[39] Graves PR , Haystead TA. Molecular biologist's guide to proteomics. Microbiol Mol Biol Rev. 2002:66(1):39–63. 11875127 10.1128/MMBR.66.1.39-63.2002 PMC120780

[40] Mouchbahani-Constance S , Sharif-Naeini R. Toxins proteomic and transcriptomic techniques to decipher the molecular evolution of venoms. Toxins. 2021:13(2):154. 33669432 10.3390/toxins13020154 PMC7920473

[41] Parker CE , Warren MR, Mocanu V. Mass spectrometry for proteomics. In: Alzate O, editors. PubMed. Boca Raton (FL): CRC Press/Taylor & Francis; 2010 Chapter 5. [Accessed 12 Mar. 2023]. Available at: https://www.ncbi.nlm.nih.gov/books/NBK56011/#_NBK56011_pubdet_ 21882443

[42] Abd El-Aziz TM , Soares AG, Stockand JD. Advances in venomics: modern separation techniques and mass spectrometry. J Chromatogr B Analyt Technol Biomed Life Sci. 2020:1160:122352. 10.1016/j.jchromb.2020.122352 PMC8174749 32971366

[43] Lomonte B , Calvete JJ. Strategies in “snake venomics” aiming at an integrative view of compositional, functional, and immunological characteristics of venoms. J Venom Anim Toxins Incl Trop Dis. 2017:23(1):26. 28465677 10.1186/s40409-017-0117-8 PMC5408369

[44] Sahyoun C , Rima M, Mattei C, Sabatier JM, Fajloun Z, Legros C. Separation and analytical techniques used in snake Venomics: a review article. Processes. 2022:10(7):1380.

[45] Daoudi K , Malosse C, Lafnoune A, Darkaoui B, Chakir S, Sabatier J, Chamot-Rooke J, Cadi R, Oukkache N. Mass spectrometry-based top-down and bottom-up approaches for proteomic analysis of the Moroccan *Buthus occitanus* scorpion venom. FEBS Open Bio. 2021:11(7):1867–1892. 10.1002/2211-5463.13143 PMC8255848 33715301

[46] Kaur P, Ghariwala V, Yeo KS, Tan HZ, Tan JCS, Armugam A, Strong PN, Jeyaseelan K. Biochemistry of envenomation. Adv. Clin. Chem. 2012:57:187–252. 10.1016/b978-0-12-394384-2.00007-3 22870591

[47] Ortiz-Prado E , Yeager J, Andrade F, Schiavi-Guzman C, Abedrabbo-Figueroa P, Terán E, Gómez-Barreno L, Simbaña-Rivera K, Izquierdo-Condoy JS. Snake anti-venom production in Ecuador: poor implementation, and an unplanned cessation leads to a call for a renaissance. Toxicon. 2021:202:90–97. 34571098 10.1016/j.toxicon.2021.09.014

[48] Gutiérrez JM , Calvete JJ, Habib AG, Harrison RA, Williams DJ, Warrell DA. Snakebite envenoming. Nat Rev Dis Primers. 2017:3(1):17063. 28905944 10.1038/nrdp.2017.63

[49] Laustsen A , Solà M, Jappe E, Oscoz S, Lauridsen L, Engmark M. Biotechnological trends in spider and scorpion antivenom development. Toxins. 2016:8(8):226. 27455327 10.3390/toxins8080226 PMC4999844

[50] Potet J , Beran D, Ray N, Alcoba G, Habib AG, Iliyasu G, Waldmann B, Ralph R, Faiz MA, Monteiro WM, et al. Access to antivenoms in the developing world: a multidisciplinary analysis. Toxicon X. 2021:12:100086. 34786555 10.1016/j.toxcx.2021.100086 PMC8578041

[51] Meier J , White J. Handbook of clinical toxicology of animal venoms and poisons. 1st ed. Bocage Raton, Florida: CRC Press; 2008 pp. 205–219

[52] Theakston RDG , Warrell DA. Anti-venoms: a list of hyperimmune sera currently available for the treatment of envenoming by bites and stings. Toxicon. 1991:29(12):1419–1470. 1801323 10.1016/0041-0101(91)90002-9

[53] Abroug F , ElAtrous S, Nouria S, Haguiga H, Touzi N, Bouchoucha S. Serotherapy in scorpion envenomation: a randomised controlled trial. Lancet. 1999:354(9182):906–909. 10489950 10.1016/s0140-6736(98)12083-4

[54] Utkin YN . Animal venom studies: current benefits and future developments. World J Biol Chem. 2015:6(2):28–33. 26009701 10.4331/wjbc.v6.i2.28 PMC4436903

[55] Zoccal KF , Sorgi CA, Hori JI, Paula-Silva FWG, Arantes EC, Serezani CH, Zamboni DS, Faccioli LH. Opposing roles of LTB4 and PGE2 in regulating the inflammasome-dependent scorpion venom-induced mortality. Nat Commun. 2016:7(1):1–13. 10.1038/ncomms10760 PMC4766425 26907476

[56] Baradaran M , Pashmforoosh N. Peptides with diverse functions from scorpion venom: a great opportunity for the treatment of a wide variety of diseases. Iran Biomed J. 2023:27(2 & 3):84–99. 37070616 10.52547/ibj.3863 PMC10314758

[57] Chen N , Xu S, Zhang Y, Wang F. Animal protein toxins: origins and therapeutic applications. Biophys Rep. 2018:4(5):233–242. 30533488 10.1007/s41048-018-0067-x PMC6245134

[58] Xia Z , He D, Wu Y, Kwok HF, Cao Z. Scorpion venom peptides: molecular diversity, structural characteristics, and therapeutic use from channelopathies to viral infections and cancers. Pharmacol Res. 2023:197:106978–106978. 37923027 10.1016/j.phrs.2023.106978

[59] Shah PT , Ali F, Noor-ul-Huda SQ, Ahmed S, Haleem K, Tauseef I, Mujaddad-ur-Rehman AH, Malik AA, Ramzan R, Khan I. Scorpion venom: a poison or a medicine-mini review. Indian J Geo Mar Sci. 2018:47(04):773–778.

[60] Kesavan K , Ratliff J, Johnson EW, Dahlberg W, Asara JM, Misra P, Frangioni JV, Jacoby DB. Annexin A2 is a molecular target for TM601, a peptide with tumor-targeting and anti-angiogenic effects. J Biol Chem. 2010:285(7):4366–4374. 20018898 10.1074/jbc.M109.066092 PMC2836041

[61] Ojeda PG , Wang CK, Craik DJ. Chlorotoxin: structure, activity, and potential uses in cancer therapy. Biopolymers. 2016:106(1):25–36. 26418522 10.1002/bip.22748

[62] De Oliveira A , Soares AM, Da Silva SL. Why to study peptides from venomous and poisonous animals. Int J Pept Res Ther. 2023:29(5):1–39.

[63] Rjeibi I , Mabrouk K, Mosrati H, Berenguer C, Mejdoub H, Villard C, Laffitte D, Bertin D, Ouafik L, Luis J, et al. Purification, synthesis and characterization of AaCtx, the first chlorotoxin-like peptide from *Androctonus australis* scorpion venom. Peptides. 2011:32(4):656–663. 21262299 10.1016/j.peptides.2011.01.015

[64] Salem ML , Shoukry NM, Teleb WK, Abdel-Daim MM, Abdel-Rahman MA. In vitro and in vivo antitumor effects of the Egyptian scorpion *Androctonus amoreuxi* venom in an Ehrlich ascites tumor model. Springerplus. 2016:5(1):570. 27247867 10.1186/s40064-016-2269-3 PMC4864766

[65] Almaaytah A , Tarazi S, Mhaidat N, Al-Balas Q, Mukattash TL. Mauriporin, a novel cationic α-helical peptide with selective cytotoxic activity against prostate cancer cell lines from the venom of the scorpion *Androctonus mauritanicus*. Int J Pept Res Ther. 2013:19(4):281–293.

[66] Khamessi O , Ben Mabrouk H, ElFessi-Magouri R, Kharrat R. RK1, the first very short peptide from *Buthus occitanus tunetanus* inhibits tumor cell migration, proliferation and angiogenesis. Biochem Biophys Res Commun. 2018:499(1):1–7. 29366787 10.1016/j.bbrc.2018.01.133

[67] Du Q , Hou X, Wang L, Zhang Y, Xi X, Wang H, Zhou M, Duan JA, Wei M, Chen T, et al. AaeAP1 and AaeAP2: novel antimicrobial peptides from the venom of the scorpion, Androctonus aeneas: structural characterisation, molecular cloning of biosynthetic precursor-encoding cDNAs and engineering of analogues with enhanced antimicrobial and anticancer activities. Toxins. 2015:7(2):219–237. 25626077 10.3390/toxins7020219 PMC4344621

[68] Crest M , Jacquet G, Gola M, Zerrouk H, Benslimane A, Rochat H, Mansuelle P, Martin-Eauclaire M-F. Kaliotoxin, a novel peptidyl inhibitor of neuronal BK-type Ca(2+)-activated K+ channels characterized from *Androctonus mauretanicus mauretanicus* venom. J Biol Chem. 1992:267(3):1640–1647. 1730708

[69] Moerman L , Bosteels S, Noppe W, Willems J, Clynen E, Schoofs L, Thevissen K, Tytgat J, Van Eldere J, van der Walt J, et al. Antibacterial and antifungal properties of α-helical, cationic peptides in the venom of scorpions from southern Africa. Eur J Biochem. 2002:269(19):4799–4810. 12354111 10.1046/j.1432-1033.2002.03177.x

[70] Khattabi A , Soulaymani-Bencheikh R, Achour S, Salmi LR, Scorpion Consensus Expert Group. Classification of clinical consequences of scorpion stings: consensus development. Trans R Soc Trop Med Hyg. 2011:105(7):364–369. 21601228 10.1016/j.trstmh.2011.03.007

[71] el Hidan MA , Touloun O, el Hiba O, Boumezzough A. Pathophysiological and neurobehavioral injuries in mice experimentally envenomed with *Androctonus liouvillei* (Pallary, 1928) scorpion venom. Exp Toxicol Pathol. 2016:68(2-3):133–141. 26651916 10.1016/j.etp.2015.11.005

[72] Ward MJ , Ellsworth SA, Nystrom GS. A global accounting of medically significant scorpions: epidemiology, major toxins, and comparative resources in harmless counterparts. Toxicon. 2018:151:137–155. 30009779 10.1016/j.toxicon.2018.07.007

[73] Sousa P , Arnedo MA, Harris DJ. Updated catalogue and taxonomic notes on the old-world scorpion genus Buthus leach, 1815 (Scorpiones, Buthidae). Zookeys. 2017:686(686):15–84. 10.3897/zookeys.686.12206 PMC5672565 29200915

[74] Das B , Saviola AJ, Mukherjee AK. Biochemical and proteomic characterization, and pharmacological insights of Indian red scorpion venom toxins. Front Pharmacol. 2021:12:1–13. 10.3389/fphar.2021.710680 PMC8505525 34650430

[75] Newlands G . Review of southern African scorpions and scorpionism. S Afr Med J. 1978:54(15):613–615. 741262

[76] Moradi M , Solgi R, Vazirianzadeh B, Tanzadehpanah H, Saidijam M. Scorpion venom and its components as new pharmaceutical approach to cancer treatment, a systematic review. Int J Pharm Sci Res. 2018:9(7):2604–2615.

[77] Nafie MS , Abdel Daim MM, Ali N, Nabil ZI, Tantawy MA, Abdel-Rahman MA. Antitumor efficacy of the Egyptian scorpion venom *Androctonus australis*: In vitro and in vivo study. J Basic Appl Zool. 2020:81(1):1–10.

